# Disturbed flow impairs MerTK-mediated efferocytosis in aortic endothelial cells during atherosclerosis

**DOI:** 10.7150/thno.93036

**Published:** 2024-03-31

**Authors:** Jinzi Wu, Shijie Liu, Oishani Banerjee, Hang Shi, Bingzhong Xue, Zufeng Ding

**Affiliations:** Department of Biology, Georgia State University, Atlanta, GA, 30303, USA.

**Keywords:** Disturbed flow, MerTK, efferocytosis, atherosclerosis, RNA-seq, proteomics

## Abstract

**Background:** MER proto-oncogene tyrosine kinase (MerTK) is a key receptor for efferocytosis, a process for the clearance of apoptotic cells. MerTK is mainly expressed in macrophages and immature dendritic cells. There are very limited reports focused on MerTK biology in aortic endothelial cells (ECs). It remains unclear for the role of blood flow patterns in regulating MerTK-mediated efferocytosis in aortic ECs. This study was designed to investigate whether endothelial MerTK and EC efferocytosis respond to blood flow patterns during atherosclerosis.

**Methods:** Big data analytics, RNA-seq and proteomics combined with our *in vitro* and *in vivo* studies were applied to reveal the potential molecular mechanisms. Partial carotid artery ligation combined with AAV-PCSK9 and high fat diet were used to set up acute atherosclerosis in 4 weeks.

**Results:** Our data showed that MerTK is sensitive to blood flow patterns and is inhibited by disturbed flow and oscillatory shear stress in primary human aortic ECs (HAECs). The RNA-seq data in HAECs incubated with apoptotic cells showed that d-flow promotes pro-inflammatory pathway and senescence pathway. Our *in vivo* data of proteomics and immunostaining showed that, compared with WT group, *MerTK^-/-^* aggravates atherosclerosis in d-flow areas through upregulation of endothelial dysfunction markers (e.g. IL-1β, NF-κB, TLR4, MAPK signaling, vWF, VCAM-1 and p22^phox^) and mitochondrial dysfunction. Interestingly, *MerTK^-/-^* induces obvious abnormal endothelial thickening accompanied with decreased endothelial efferocytosis, promoting the development of atherosclerosis.

**Conclusions:** Our data suggests that blood flow patterns play an important role in regulating MerTK-mediated efferocytosis in aortic ECs, revealing a new promising therapeutic strategy with EC efferocytosis restoration to against atherosclerosis.

## Introduction

Atherosclerosis is the most common underlying cause of ischemic heart disease and stroke, and it accounts for one in four deaths worldwide 1. The disease progresses slowly with chronic inflammation and a buildup of lipid laden plaques in large and medium sized arteries 1. A large body of evidence has shown that the disturbed flow (d-flow) pattern in arterial branch points and curvatures causes the preferential localization of atherosclerotic lesions, while regions with steady laminar flow (s-flow) where high shear stress exists are protected against atherosclerosis 2. Aortic endothelial cells (ECs) are the main component of the inner lining of the blood vessel wall and directly exposed to blood flow, playing important roles in vascular function in response to various chemical and mechanical stimuli 3.

MER proto-oncogene tyrosine kinase (MerTK) is a member of the TAM (Tyro3, Axl and MerTK) receptor family and plays a key role in the efficient clearance of apoptotic cells, a process called efferocytosis 4. Efferocytosis is accomplished by either professional phagocytes such as macrophages and immature dendritic cells with a relatively faster ability in engulfment of apoptotic cells, or non-professional phagocytes such as ECs and epithelial cells for clearance of neighboring dying cells 5. Efficient efferocytosis promotes differentiation of phagocytes into an anti-inflammatory phenotype, whereas impaired efferocytosis leads to the detrimental accumulation of apoptotic cells 6. Impaired efferocytosis in advanced atherosclerosis can lead to the buildup of dead cells and cellular debris of necrotic core formation, which can trigger plaque rupture and acute thrombotic cardiovascular events 7.

We recently reported that primary aortic ECs have high ability to perform efferocytosis and EC efferocytosis plays important roles in vascular aging 8. However, the previous studies did not show the direct link between blood flow patterns and EC efferocytosis as well as the interaction between blood flow patterns and MerTK expression. The role of MerTK in d-flow-mediated endothelial dysfunction and its implications for the pathological process of atherosclerosis, is also unknown. This study was designed to address these unknown questions and determine whether these processes can be centers of new therapeutic strategies against atherosclerosis, especially for cases resistant to current treatments.

## Methods

### Animals

Wild-type (WT) and *MerTK^-/-^* mice were purchased from the Jackson Laboratories (Sacramento, CA, USA) and housed in the Division of Laboratory Animal Medicine at our institution. All experimental procedures were performed in accordance with protocols approved by the Institutional Animal Care and Use Committee and conformed to the Guidelines for the Care and Use of Laboratory Animals published by the US National Institutes of Health. Partial carotid ligation (PCL) was done as described in DOI:10.3791/1861 9. Briefly, anesthesia was induced with isoflurane (3-4% for induction, 1-3% for maintenance) to the male mice. After disinfection, a ventral midline incision was made in the neck, and the left common carotid artery will be exposed by blunt dissection. The external carotid artery, internal carotid artery, and occipital artery were ligated with 6-0 silk suture; the superior thyroid artery was kept open. The right common carotid artery, with no ligation, served as a built-in control. To accelerate atherosclerosis, one week prior to PCL ligation, some mice were injected with a single dose of AAV8-PCSK9 particles (1×10^11^) combined with a high fat diet (HFD, TD.88137, Envigo) or AAV control via the tail vein. Mice were monitored until recovery in a chamber at 37 °C. For additional pain relief, 3 doses of meloxicam at 4µ/g were given by subcutaneous injection prior or after PCL every 24 h.

### Tissue collection

After mice were euthanized by CO_2_ asphyxiation at the 4-week endpoint, the right carotid artery (RCA) and left carotid artery (LCA) as well as aortic arch and thoracic aorta were carefully dissected from surrounding tissue, then fixed with 10% neutral buffered formalin solution (Sigma, HT501128) and embedded in paraffin or stored at -80 °C for further molecular, histological and immunohistochemical analyses.

### Cells

Primary human aortic ECs (HAECs) and the human Jurkat cell line were purchased from ATCC (Manassas, VA, USA). HAECs were cultured with Vascular Cell Basal Medium (ATCC, PCS-100-030) and Endothelial Cell Growth Kit (ATCC, PCS-100-041). Jurkat cells were cultured in RPMI-1640 medium (ATCC, 30-2001) with 10% Fetal Bovine Serum (FBS, ATCC 30-2020).

### Efferocytosis evaluation

EC efferocytosis was analyzed by a protocol modified from a published method 10. Briefly, ECs were labeled with PKH26-Red (2 μM; Sigma-Aldrich) and were plated (1 × 10^6^ cells/well) on a 6-well cell culture plate for 24 h or allowed to reach confluence. Jurkat cells, an acute T-cell leukemia cell line widely used for efferocytosis studies in cardiovascular disease, were labeled with PKH67-Green (2 μM; Sigma-Aldrich) and exposed to UV light (254 nm, UVP) for 5 min to induce apoptosis; they then were incubated at 37 °C with 5% CO_2_ for 1 h. EC medium was replaced with medium containing apoptotic Jurkat cells to achieve a cell ratio of 3:1, or as indicated for apoptotic Jurkat cells/ECs. After incubation for 1 h at 37 °C, the ECs were washed twice with cold PBS. The percentage of ECs labeled with PKH67-Green from engulfing apoptotic cells were quantified with fluorescence microscopy.

### Western blot

Protein was extracted with RIPA Lysis Buffer System (Santa Cruz, CA, USA) and loaded onto Mini-PROTEAN® TGX™ Precast Gels (Bio-rad, CA, USA) for electrophoresis. The size-separated proteins were then transferred to Hybond ECL Nitrocellulose Membranes (GE Healthcare, NJ, USA). After blocking with 5% BSA buffer for 1 h, the membranes were incubated with primary antibody recognizing either MerTK (Santa Cruz), NLRP3 (Cell Signaling), pro-IL-1β (Cell Signaling), cleaved IL-1β (Cell signaling), TNF-α (Cell Signaling), NF-κB (Cell Signaling), eNOS (Cell Signaling), Gas 6 (Cell Signaling), TGF-β (Cell Signaling), or β-actin (Abcam) at 1:1000 dilution overnight at 4 °C. After washing with PBS containing 0.1% Tween-20, membranes were incubated with secondary antibody targeting either anti-rabbit (Abcam, ab6721) or anti-mouse (Abcam, ab6708) at 1:4000 dilution for 1 h and signals were detected with Pierce ECL western blotting substrate (Thermo Fisher Scientific, MA, USA). Intensity quantification of the bands was performed with Image J software and normalized to β-actin.

### Immunofluorescent staining

Immunofluorescent staining was performed according to the protocol of 'Immunofluorescent Staining of Paraffin-Embedded Tissue (Novus Biologicals)'. The information of antibodies is shown as follows: cleaved IL-1β (Cell Signaling), Caspase-3 (Santa Cruz), Phospho-p70s6k (Cell Signaling), Phospho-EIF2α (Cell Signaling), EIF3A (Cell Signaling), vWF (Abcam), VCAM-1 (Cell Signaling) and p22^phox^ (Cell Signaling).

### RNA sequencing (RNA-seq) in HAECs

RNA was extracted from five randomly selected samples based on seven prepared samples in each group using Quick DNA/RNA miniprep kit (Zymo Research, CA, USA). The quality of RNA was assessed using Qubit RNA Broad Range Assay and fragment analyzer (Agilent, CA, USA). RNA libraries were prepared using TruSeq Stranded Total RNA Kit (Illumina, CA, USA) and then sequenced on a NovaSeq 6000 system with a SP 200-cycle flow cell. RNA-seq reads were quality-checked, trimmed, and aligned to the GRCm39 reference genome (accession: GCA_000001635.9) using the Nextflow RNAseq pipeline, nf-core/rnaseq (version 3.4 available at DOI 10.5281/zenodo.1400710). The resulting gene counts were transformed to log2 counts per million (CPM) 11. Genes with a low expression were filtered out and libraries were normalized by trimmed mean of M-values 12. The Limma R package was used to calculate differential expression among genes 13. Log2 fold change values were calculated for each sample compared to control. Genes with an absolute fold change > 2 were considered significant.

### Proteomics measurements in carotid artery

Proteomics in the RCA or LCA (n = 3 per group) were performed with Orbitrap Exploris 480 Mass Spectrometer (Thermo) at the IDeA National Resource for Quantitative Proteomics at our institution using a data independent acquisition (DIA) protocol. Briefly, proteins were reduced, alkylated, and purified by chloroform/methanol extraction prior to digestion with sequencing grade modified porcine trypsin (Promega). Tryptic peptides were then separated by reverse phase XSelect CSH C18 2.5 um resin (Waters) on an in-line 150 x 0.075 mm column using an UltiMate 3000 RSLCnano system (Thermo Fisher Scientific). Peptides were eluted using a 60 min gradient from 98:2 to 65:35 buffer A:B ratio (A = 0.1% formic acid and 0.5% acetonitrile; B = 0.1% formic acid and 99.9% acetonitrile). Eluted peptides were ionized by electrospray (2.2 kV) followed by mass spectrometric analysis. To assemble a chromatogram library, six gas-phase fractions were acquired on the Orbitrap Exploris with 4 m/z DIA spectra (4 m/z precursor isolation windows at 30,000 resolution, normalized AGC target 100%, maximum inject time 66 ms) using a staggered window pattern from narrow mass ranges using optimized window placements. Precursor spectra were acquired after each DIA duty cycle, spanning the m/z range of the gas-phase fraction (i.e., 496-602 m/z, 60,000 resolution, normalized AGC target 100%, maximum injection time 50 ms). For wide-window acquisitions, the Orbitrap Exploris was configured to acquire a precursor scan (385-1015 m/z, 60,000 resolution, normalized AGC target 100%, maximum injection time 50 ms) followed by 50x 12 m/z DIA spectra (12 m/z precursor isolation windows at 15,000 resolution, normalized AGC target 100%, maximum injection time 33 ms) using a staggered window pattern with optimized window placements. Precursor spectra were acquired after each DIA duty cycle.

Following data acquisition, data were searched using an empirically corrected library and a quantitative analysis was performed to obtain a comprehensive proteomic profile. Proteins were identified and quantified using EncyclopeDIA 14 and visualized with Scaffold DIA using 1% false discovery thresholds at both the protein and peptide level. Protein MS2 exclusive intensity values were assessed for quality using ProteiNorm 15. The data were normalized by cyclic loss to perform statistical analysis using linear models for microarray data (limma) with empirical Bayes (eBayes) smoothing to the standard errors 16. Proteins with an FDR adjusted p-value < 0.05 and a fold change > 2 were considered significant. The proteomics data were analyzed by Ingenuity Pathway Analysis (IPA, Qiagen) and IDeA National Resource (idearesourceproteomics.org).

### Statistical analysis

Statistical analysis was performed with GraphPad Prism 9.4.1. Data were summarized as the mean ± standard deviation (SD). An unpaired Student's *t*-test was used to determine statistical significance. Comparisons between multiple points were subjected to one-way ANOVA with Tukey's or Dunnett's post hoc tests; *P* <0 .05 was considered significant.

## Results

### D-flow inhibits MerTK expression and induces endothelial dysfunction

**Figure 1:**
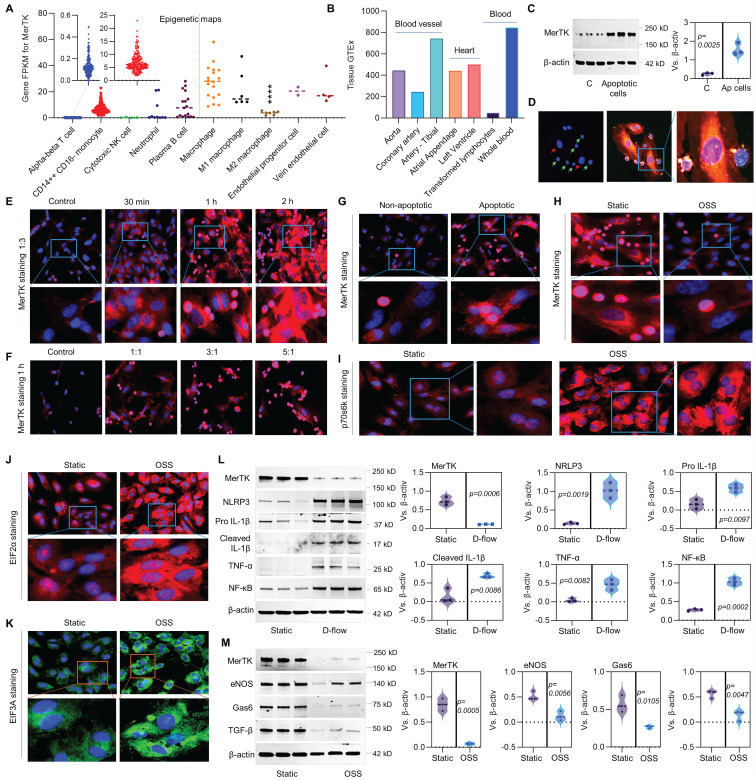
** Efferocytosis in ECs and the role of d-flow in regulation of MerTK.** (**A**) Epigenetic maps for MerTK expression in hematopoietic cells from healthy individuals based on IPA gene FPKM. (**B**) MerTK expression in vascular system based on IPA tissue GTEx. (**C**) Western blotting for MerTK expression in HAECs incubated with apoptotic Jurkat cells for 1 h. (**D**) Efferocytosis in HAECs incubated with apoptotic Jurkat cells for 1 h. Green cells: apoptotic Jurkat cells labeled with PKH67 Green Fluorescent Cell Linker Kit (PKH67GL-1KT, Sigma); Green/red small round cells: apoptotic Jurkat cells that were engulfed by HAECs; Large red cells: HAECs labeled with PKH26 Red Fluorescent Cell Linker Kit (PKH26GL-1KT, Sigma) according to the provided protocol. (**E**-**F**) Immunostaining for MerTK expression in HAECs incubated with apoptotic Jurkat cells for 30 min, 1 h or 2 h; or incubated with apoptotic Jurkat cells with a different ratio of 1:1, 3:1 or 5:1. (**G**) Immunostaining for MerTK expression in HAECs incubated with non-apoptotic or apoptotic Jurkat cells with a ratio of 3:1. (**H**-**K**) Efferocytosis and expression of p70s6k, EIF2α and EIF3A in HAECs subjected to OSS or in a static condition. (**L**) Effect of d-flow in regulation of MerTK and endothelial function in a step-flow chamber (12 ± 4 dynes/cm^2^ for 1 h). (**M**) Effect of OSS (± 5 dynes/cm^2^ with 1Hz in a µ-Slide I^ 0.4^ Luer for 1 h) in regulation of MerTK and endothelial function in an ibidi pump system. Statistical analyses were performed with GraphPad Prism 9.4.1 using a two-tailed unpaired t-test. Data represents mean ± SD (n=3-6).

MerTK is mainly expressed in macrophages 4-7, there are very limited studies focused on MerTK expression in ECs. Therefore, we first performed the big data analytics from Blueprint-B38-GC33 that focuses on epigenetic maps of distinct types of hematopoietic cells from healthy individuals and on their malignant leukemic counterparts. As shown in **Figure 1A**, epigenetic maps showed that MerTK is highly expressed in CD14^++^CD16^-^ monocytes, macrophages, and plasma B cells as well as endothelial progenitor cells and vein ECs. There is no significant difference of MerTK expression between macrophages, endothelial progenitor cells and vein ECs based on one-way ANOVA with Tukey's multiple comparisons test. To further investigate the potential role of MerTK in ECs, another big data analytics was performed by Genotype-Tissue Expression project (GTEx, B38-GC33) based on RNA-Seq and Affymetrix expression data from human normal tissues (**Figure 1B**). The tissue GTEx data showed that MerTK is expressed in blood vessels (e.g., aorta, coronary artery, and tibial artery), heart (e.g., atrial appendage and left ventricle), and blood (e.g., transformed lymphocytes and whole blood). Next, we studied whether apoptotic cells could activate MerTK expression and efferocytosis in aortic ECs. Human Jurkat cell is an acute T cell leukemia cell line with about 10.72 μm in diameter that has been widely used for efferocytosis studies in phagocytes 10. Our Western blotting data showed that apoptotic Jurkat cells significantly induced MerTK expression in primary human aortic ECs (HAECs, **Figure 1C**). Our immunostaining data showed that HAECs have high ability to perform efferocytosis for the clearance of apoptotic Jurkat cells, which were incubated with HAECs only for 1 h (**Figure 1D**). Interestingly, our study showed that apoptotic Jurkat cells induce MerTK expression in a time- and dose-dependent manner (**Figure 1E** and **1F**). MerTK expression increases with the increasing incubation time of apoptotic cells up to 2 h. However, MerTK expression has the highest expression with a cell ratio of 1:1 (apoptotic Jurkat cells/ECs) and it does not significantly increase with the cell ratio of 3:1 and 5:1. It has been shown that Jurkat cells abundantly express MerTK 17. To clarify if engulfed Jurkat cells are responsible for increased MerTK expression in HAECs, both non-apoptotic Jurkat cells and apoptotic Jurkat cells were incubated with HAECs (**Figure 1G**). On one hand, we found that MerTK is highly expressed in Jurkat cells that is consistent with previous study 17. On the other hand, we found that apoptotic Jurkat cells markedly activates MerTK expression in HAECs, indicating that apoptotic Jurkat cells partly account for the increased EC MerTK expression. Our data also showed that HAECs prefer to degrade apoptotic Jurkat cells compared to non-apoptotic Jurkat cells.

To investigate the role of d-flow in MerTK expression in HAECs, a step-flow chamber that generates d-flow characterized by low and oscillatory wall shear stress 18 and an ibidi pump system that generates low and oscillatory shear stress (OSS) were applied. Our data showed that OSS significantly inhibits efferocytosis in HAECs compared to static group (**Figure 1H**). p70 S6 kinase (p70S6K) activation is an important process for vascular remodeling in pathophysiological conditions 19. Eukaryotic translation initiation factor 2 alpha (EIF2α) plays a central role in regulating protein synthesis initiation by its phosphorylation or dephosphorylation 20. EIF2α is involved in apoptosis activation in vascular ECs exposed to endoplasmic reticulum stress 21. Eukaryotic translation initiation factor 3a (EIF3A) regulates protein synthesis and increased levels of EIF3A have been shown in advanced atherosclerotic lesions 22. Consistently, our study showed that, compared to static conditions, OSS markedly induces expression of p70S6K, EIF2α and EIF3A (**Figure 1I-1K**). In step-flow chamber, d-flow markedly inhibited MerTK expression while induced endothelial dysfunction, indicating increased expression of pro-inflammatory signaling such as NLRP3 inflammasome (NLRP3, pro-IL-1β, and cleaved IL-1β), TNF-α, and NF-κB (**Figure 1L**). Consistently, OSS markedly inhibited MerTK expression and induced endothelial dysfunction shown as decreased expression of endothelial nitric oxide synthase (eNOS), Gas 6 (ligand of MerTK), and transforming growth factor-β (TGF-β, anti-inflammatory cytokine) (**Figure 1M**).

### RNA-seq in HAECs incubated with the apoptotic cells in a step-flow chamber

**Figure 2:**
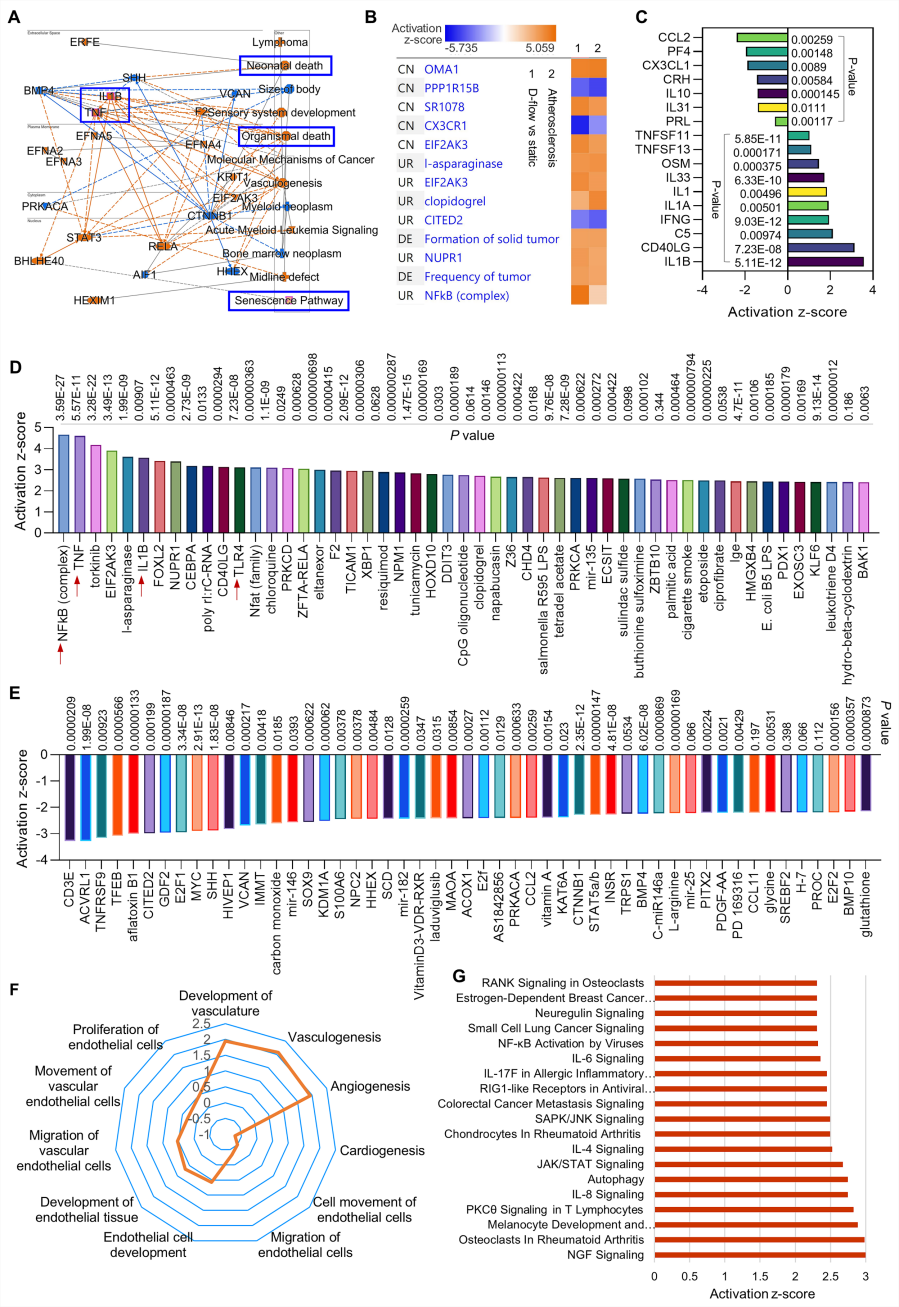
** RNA-seq in HAECs that are exposed to d-flow and incubated with apoptotic cells.** (**A**) Graphical summary of RNA-seq data in HAECs exposed to d-flow or static conditions (orange: upregulated; blue: downregulated). (**B**) IPA comparative analysis between d-flow vs static and atherosclerosis based on activation z-score. (**C**) Expression of cytokines or chemokines in HAECs based on activation z-score. (**D-E**) Top 50 representative proteins upregulated or downregulated by d-flow in HAECs, based on activation z-score. (**F**) Endothelial function evaluation based on activation score. (**G**) Top ingenuity canonical pathways in HAECs based on activation z-score. HAECs were kept in static conditions or subjected to step-flow chamber with physiological laminar shear stress (12 ± 4 dynes/cm^2^) for 24 h and then were incubated for 1 h with PKH67-green linker-labeled apoptotic Jurkat cells at 3:1 ratio (apoptotic cells:HAECs). For HAECs in d-flow area, RNA was extracted from 5 randomly selected frozen specimens per experimental group and RNA-seq was done at UAMS Genomics Core using Next Generation Sequencing.

To further investigate the role of d-flow in regulating endothelial function, RNA-seq in HAECs exposed to d-flow or in a static condition for 24 h, and subsequently incubated with apoptotic Jurkat cells for another 1 h. **Figure 2A** shows a graphical summary of the RNA-seq data in HAECs. Compared with static control, d-flow upregulates pro-inflammatory cytokines (e.g., TNF- tumor necrosis factor and IL-1β), cell death pathway, and the senescence pathway; all contribute to endothelial dysfunction. Because impaired efferocytosis and endothelial dysfunction are associated with atherosclerosis 6, we compared the group of d-flow vs. static with public shared RNA-seq data of atherosclerosis based on Qiagen IPA database.

We found several novel activation pathways between d-flow and atherosclerosis (**Figure 2B**), such as Zn^2+^-dependent inner mitochondrial membrane metalloendopeptidase OMA1, SR 1078 (a selective agonist of RORα and RORγ) and EIF2AK3 (eukaryotic translation initiation factor 2 alpha kinase 3). There are also several novel inhibition pathways, such as PPP1R15B (a negative regulator of proteostasis), CX3CR1 (C-X3-C Motif Chemokine Receptor 1, a novel inflammatory adipose chemokine system) and CITED2 (Cbp/p300 interacting transactivator with Glu/Asp rich carboxy-terminal domain 2). For the cytokines and chemokines analysis (**Figure 2C**), our data showed that d-flow significantly inhibits expression of CCL2 (C-C motif chemokine ligand 2), PF4 (platelet factor 4) and IL-10 (anti-inflammatory cytokine), while induces expression of IL-1β (pro-inflammatory cytokine), CD40LG (CD40 Ligand) and IFN‐γ (interferon gamma, a pro-inflammatory cytokine). For the upstream signaling analysis, we identified the 50 most activated genes in HAECs exposed to d-flow compared with static conditions (**Figure 2D**). Activation z-scores analysis indicated that d-flow markedly activates the NF-κB complex, TNF, and toll-like receptor 4 (TLR4) pathways. **Figure 2E** shows the top 50 most downregulated genes in HAECs of d-flow vs. static control, including CD3E (cd3 epsilon subunit of T-cell receptor complex), ACVRL1 (activin receptor like-protein 1, a member of the TGF-β receptor family), E2F1 (E2F transcription factor 1, involved in cell cycle, DNA synthesis and replication, and DNA damage and repair). For the endothelial function analysis (**Figure 2F**), we found that d-flow promotes development of vasculature, vasculogenisis and angiogenesis while inhibits cardiogenesis, EC movement and EC migration. **Figure 2G** shows top ingenuity canonical pathways in HAECs exposed to d-flow based on activation z-score, including increased NGF (nerve growth factor) signaling, IL-8 signing, JAK/STAT signaling, and NF-κB activation signaling.

### MerTK is sensitive to d-flow *in vivo* and regulates endothelial efferocytosis and endothelial dysfunction

**Figure 3:**
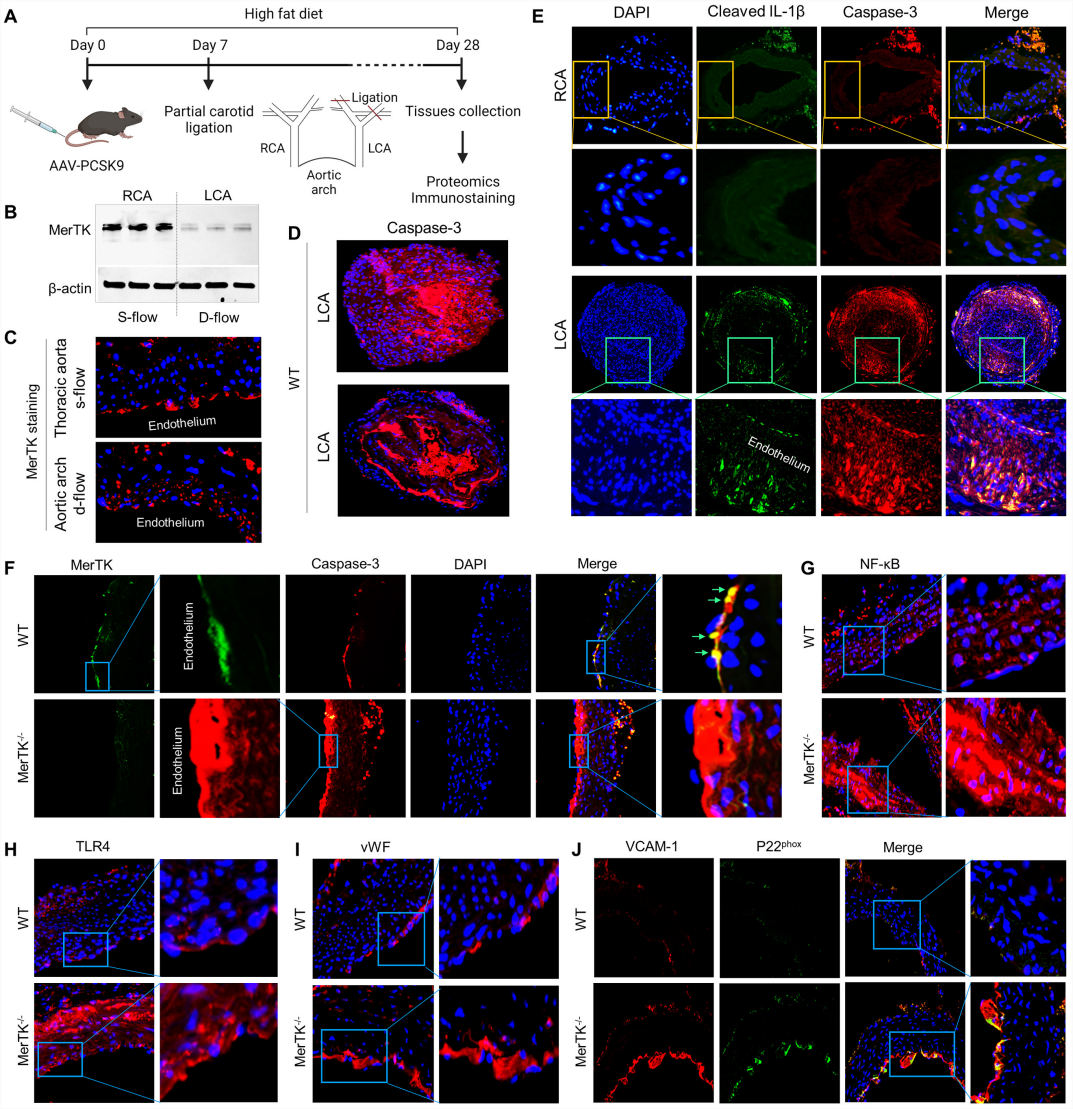
** PCL surgery and atherosclerosis model in WT and *MerTK^-/-^* mice.** (**A**) Schematic diagram for acute atherosclerosis model combined with PCL surgery and AAV8-PCSK9 injection. (**B**) Westen blotting for MerTK expression in RCA or LCA from WT mice with PCL surgery. (**C**) Immunostaining for MerTK in the straight section of thoracic aorta and aortic arch. (**D**) Immunostaining for Caspase-3 in LCA from WT mice with PCL surgery. (**E**) Representative immunostaining for cleaved IL-1β and Caspase-3 in RCA and LCA from *MerTK^-/-^* and WT mice, respectively. (**F**-**J**) Immunostaining for MerTK, Caspase-3, NF-kB, TLR4, vWF, VACAM-1 and p22^phox^ in aortic arch from *MerTK^-/-^* and WT mice. Mice (n=5-7) were injected with one dose of AAV8-PCSK9, and PCL surgery was performed 1-week later. The mice were fed HFD for 4 weeks

PCL is a widely used model to generate d-flow in the left carotid artery (LCA), accelerating rapid endothelial dysfunction and atherosclerosis; while the right carotid artery (RCA) without PCL is a built-in control 23. The function of AAV8-PCSK9 is the same as *LDLr^-/-^* that induces hypercholesterolemia and accelerates atherosclerosis 24. **Figure 3A** shows our atherosclerosis model combined AAV8-PCSK9 with high fat diet (HFD) that is an effective and time-saving approach to set up rapid atherosclerosis in 4 weeks. First, we studied whether MerTK is regulated by blood flow patterns *in vivo*. Our Western blotting data shows that MerTK is significantly inhibited in LCA rich in d-flow compared with built-in control RCA with s-flow, indicating that MerTK is sensitive to blood flow patterns *in vivo* (**Figure 3B**). Consistently, MerTK expression is much higher in the straight section of thoracic aorta (s-flow) than that in aortic arch (d-flow, **Figure 3C**). It is noted that, in aortic arch, endothelium areas where is lack of MerTK appeared to exhibit increased MerTK expression possibly in macrophages in the arterial media, indicating the increased apoptotic cells that is associated with vascular dysfunction. **Figure 3D** shows the immunostaining of apoptotic marker caspase-3 in LCA from WT mice, an indicator for the efficiency of PCL-induced atherosclerosis model and accumulation of apoptosis in atherosclerotic plaque. Interestingly, **Figure 3D** also shows the formation of aortic dissection (lower panel), suggesting that PCL may be a potential model for aortic aneurysm and dissection. **Figure 3E** shows that there was no plaque formation in RCA while obvious plaque formation was found in LCA from both WT and *MerTK^-/-^* mice. **Figure 3E** also shows that, compared with RCA, both cleaved IL-1β (green) and caspase-3 (red, apoptotic marker) were significantly increased in LCA with plaque area. Second, to investigate whether it exists endothelial efferocytosis *in vivo*, double immunostaining for MerTK and Caspase-3 in aortic arch were performed. In WT mice, MerTK is mainly expressed in endothelium and endothelial efferocytosis was detected (**Figure 3F**). Compared with WT mice, Caspase-3 expression in endothelium is much higher and there is almost no endothelial efferocytosis from *MerTK^-/-^* mice. An obvious formation of aortic dissection was also found in *MerTK^-/-^* mice. Finally, we investigated expression of endothelial dysfunction markers in aortic arch from both WT and *MerTK^-/-^* mice such as NF-kB, TLR4, vWF (von Willebrand factor, is known to contribute to atherosclerosis) 25, VACAM-1 (vascular cell adhesion molecule 1) and NADPH oxidase subunit p22^phox^ (responsible for ROS production) 26. Our data showed that, compared with WT mice, *MerTK^-/-^* significantly induces expression of as NF-kB, TLR4, vWF, VACAM-1 and p22^phox^ (**Figure 3G-3J**). These findings raise the intriguing possibility that MerTK plays a key role in endothelial dysfunction during the development of atherosclerosis.

### Proteomics in LCA and RCA in WT and *MerTK^-/-^* mice subjected to PCL surgery

**Figure 4:**
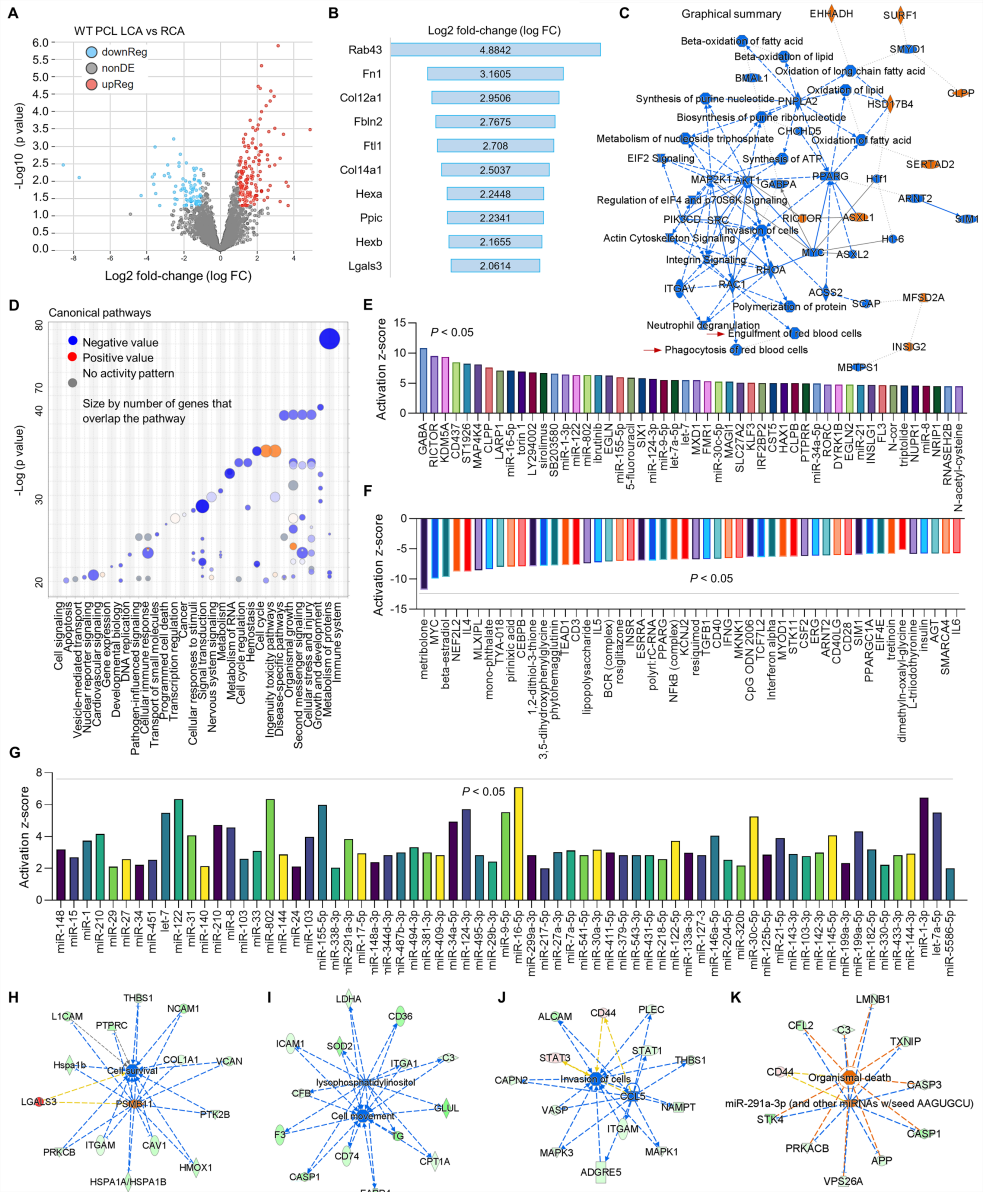
** Proteomics comparison of LCA vs. RCA in WT mice subjected to PCL surgery.** (**A**) Volcano plot illustrating differentially expressed proteins in RCA and LCA. Relative protein abundance (log2) plotted against significance level (- log10 P-value), showing significantly (p < 0.05) downregulated (blue), upregulated (red) or non-differentially expressed proteins (gray). (**B**) Top 10 activated proteins based on Log2 fold change. (**C**) Graphical summary based on IPA. (**D**) Bubble chart for canonical pathways. (**E**-**F**) Top 50 representative proteins upregulated or downregulated in LCA compared to RCA based on activation z-score. (**G**) IPA analysis for microRNA in LCA vs. RCA. (**H**-**K**) Representative vascular functions and their related signaling.

To further investigate the relationship between d-flow and MerTK in vascular function, proteomics was performed in both RCA and LCA from WT and *MerTK^-/-^* mice subjected to PCL. Firstly, we analyzed the proteomic changes in LCA compared with RCA from WT mice subjected to PCL surgery. **Figure 4A** shows a clear separation in protein profiles between LCA and RCA, including 151 upregulated proteins and 87 downregulated proteins. **Figure 4B** shows top 10 protein changes based on Log 2-fold-change with adjusted p value <0.05, such as Rab43 (Ras related protein), Fn1 (fibronectin 1) and Col12a1 (collagen 12a1). **Figure 4C** is the graphical summary, showing the inhibited engulfment and phagocytosis of red blood cells, EIF2 signaling, and synthesis of ATP while the induced ASXL1 (ASXL Transcriptional Regulator 1, for chromatin remodeling), HSD17B4 (hydroxysteroid-17-beta-dehydrogenase 4, for peroxisomal fatty acid beta-oxidation), and INS1G2 (insulin induced protein 2, associated with cardiovascular disease). **Figure 4D** is the canonical pathways that show activated pathways (e.g., cell cycle and ingenuity toxicity pathways) and inhibited pathways (e.g., immune system, cellular response to stimuli, and metabolism of RNA). Next, we analyzed the upstream pathways of upregulated (**Figure 4E**) and downregulated (**Figure 4F**) signaling based on activation z-score. It is noted that many microRNAs (miR) were activated in LCA compared to RCA, which was summarized in **Figure 4G**. Finally, we analyzed vascular functions in LCA compared to RCA, showing inhibited cell survival (**Figure 4H**), cell movement (**Figure 4I**) and invasion of cells (**Figure 4J**), while induced cell death (**Figure 4K**).

**Figure 5:**
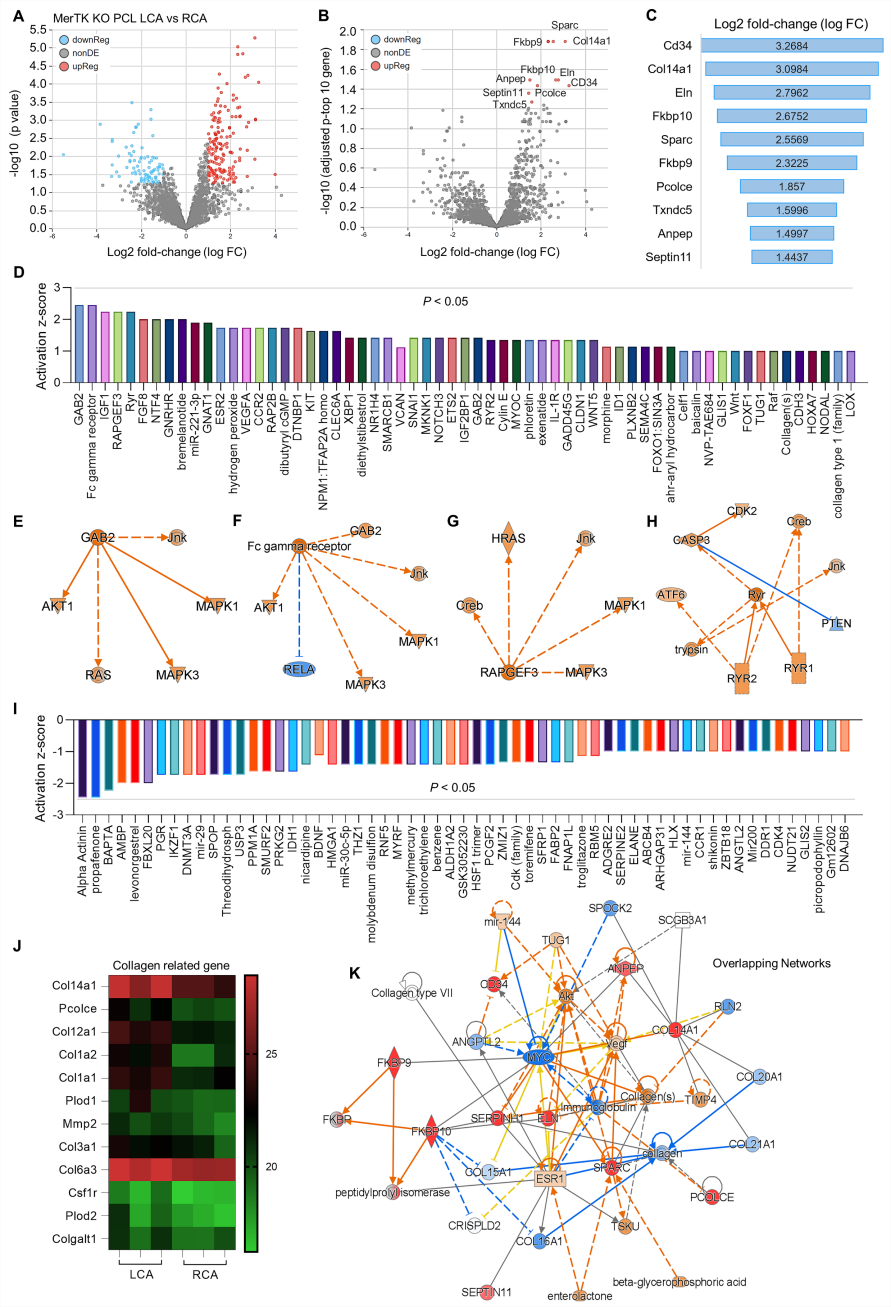
** Proteomics comparison of LCA vs. RCA in *MerTK^-/-^* mice subjected to PCL surgery.** (**A**) Volcano plot illustrating differentially expressed proteins in RCA and LCA. Relative protein abundance (log2) plotted against significance level (- log10 P-value), showing significantly (p < 0.05) downregulated (blue), upregulated (red) or non-differentially expressed proteins (gray). (**B**) Volcano plot illustration for top 10 changed proteins based on adjusted p value and Log2 fold change, which are detailed shown in (**C**). (**D**) Top 50 activated proteins based on activation z-score. (**E**-**H**) Representative activated proteins related to MAPK pathways based on activation z-score. (**I**) Top 50 inhibited proteins based on activation z-score. (**J**) Collagen-related proteins in LCA and RCA. (**K**) Overlapping networks in LCA compared to RCA.

Secondly, we analyzed proteomics in RCA and LCA in *MerTK^-/-^* mice underwent PCL surgery. **Figure 5A** shows the volcano plot illustration with a clear separation in protein files, which includes 172 upregulated proteins and 87 downregulated proteins. We further adjusted the p value to show the top 10 protein changes (**Figure 5B** and **5C**), such as CD34 (a marker of endothelial progenitor cells), Col4a1 (collagen type IV alpha 1 chain), and Eln (elastin, the major component of elastic fibers). Then we analyzed the upstream signaling with activated causal networks (**Figure 5D**), which shows the top 50 upregulated proteins in LCA compared to RCA. We found that mitogen-activated protein kinases (MAPKs) may play an important role in interaction between PCL-induced d-flow and vascular function. As shown in **Figure 5E** to **5H**, the networks based on CAB2 (chlorophyll A/B-binding protein 2), FC (fragment crystallizable) gamma receptor, RAPGEF3 (Rap Guanine Nucleotide Exchange Factor 3) and CASP3 (caspase 3, an apoptotic marker) show that MAPKs (e.g., MAPK1,3 and JNK) are activated in LCA subjected to acute d-flow. Next, we analyzed the inhibited causal networks of upstream signaling shown in **Figure 5I**. Interestingly, we noticed that there are many collagens signaling involved in protein changes LCA from both activated and inhibited causal networks (**Figure 5D** and **5I**). We summarized collagen-related proteins in both RCA and LCA (**Figure 5J**), which shows significantly induced collagens (e.g., Col14a1, Pcolce, Col12a1, Col1a2/1, Plod2) in LCA compared to RCA. Finally, we analyzed overlapping networks in LCA vs. RCA in **Figure 5K**, which shows activated pathways (e.g., collagens, AKT, and CD34) and inhibited pathways (e.g., MYC- a proto-oncogene, immunoglobulin, and other collagen subunits).

**Figure 6:**
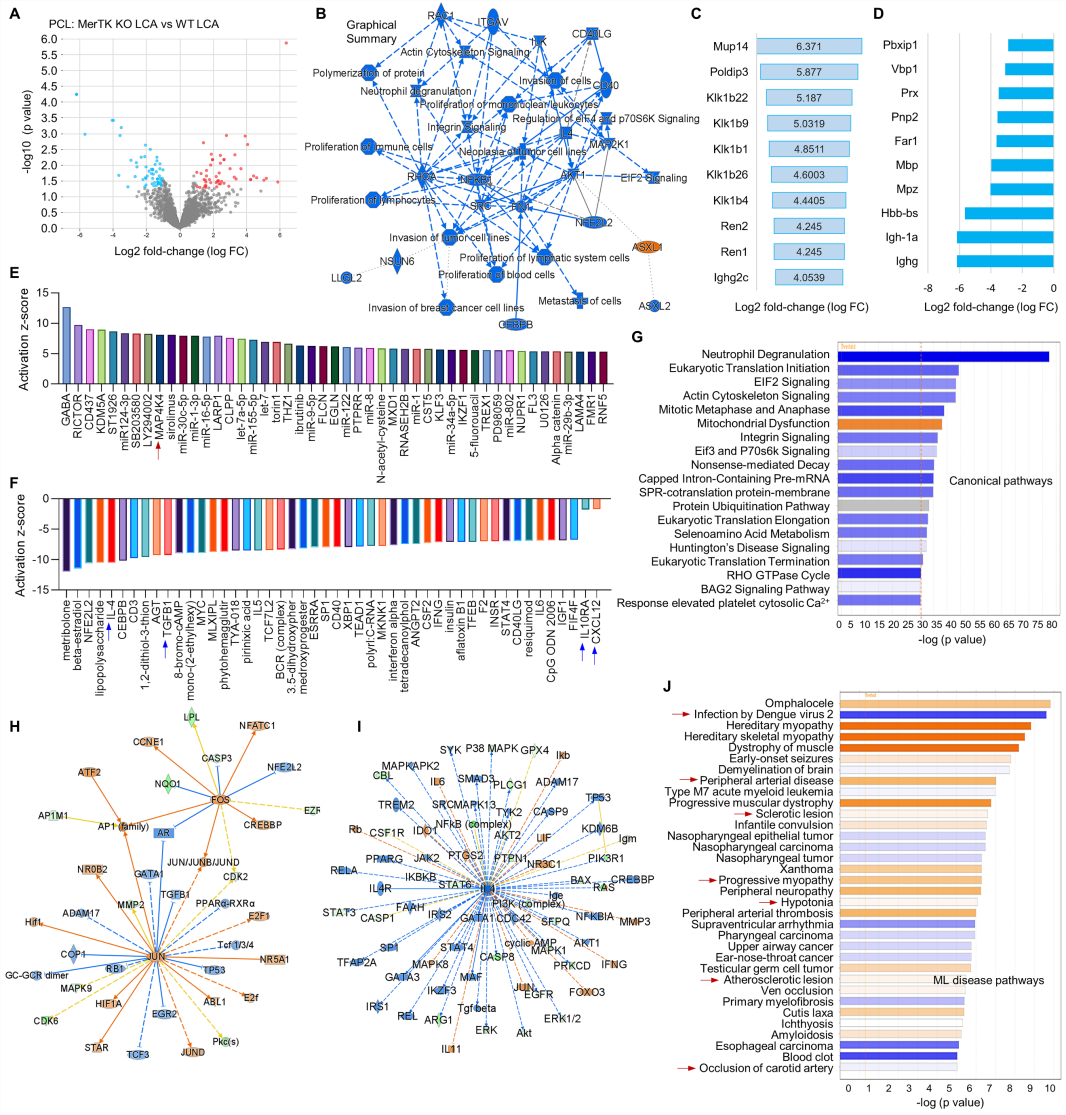
** Proteomics comparison in LCA from WT and *MerTK^-/-^* mice subjected to PCL surgery.** (**A**) Volcano plot illustrating differentially expressed proteins in LCA compared *MerTK^-/-^* with WT. Relative protein abundance (log2) plotted against significance level (- log10 P-value), showing significantly (p < 0.05) downregulated (blue), upregulated (red) or non-differentially expressed proteins (gray). (**B**) Graphical summary for the down downregulated (blue) and upregulated (red) proteins. (**C**) Top 10 upregulated proteins based on Log2 fold-change. (**D**) To 10 downregulated proteins based on Log2 fold-change. (**E**) Upstream signaling for top 50 activated proteins based on activation z-score. (**F**) Upstream signaling for top 50 inhibited proteins based on activation z-score. (**G**) Canonical pathways based on -log (p value) shown as downregulated (blue) and upregulated (red) signaling. (**H**-**I**) Representative upregulated signaling such as JUN and FOS as well as downregulated signaling such as IL-4 and IL-4R. (**J**) IPA for disease pathways in LCA compared *MerTK^-/-^* with WT.

Thirdly, we compared the changes of proteomics in LCA subjected to PCL from WT and *MerTK^-/-^* mice, indicating the role of MerTK in response to d-flow. **Figure 6A** shows the volcano plot illustration with 52 upregulated proteins and 57 downregulated proteins. **Figure 6B** shows the graphical summary. We found that, compared with WT LCA, ASXL1 (ASXL Transcriptional Regulator 1) is specifically activated while a variety of pathways were inhibited, such as proliferation of immune cells, actin cytoskeleton signaling and EIF2 signaling in *MerTK^-/-^* LCA. The role of ASXL1 in atherosclerosis remains largely unknown and EIF2 downregulation promotes atherosclerosis, indicating that ASXL1 and EIF2 may play novel roles in hemodynamics-mediated EC efferocytosis, which needs to be further validated. **Figure 6C** and **6D** show top 10 activated signaling and top 10 inhibited signaling based on Log2 fold change excluding MerTK, respectively. These signaling such as Podip3 (polymerase delta-interacting protein 3), Klk1b22 (kallikrein 1-related peptidase b22), lghg (Immunoglobulin heavy constant gamma) may represent the novel mechanisms in EC efferocytosis. Adhesion molecules, pro- or anti-inflammatory factors and chemokines play important roles in atherosclerosis.^1^ For adhesion molecules, it has been shown that MerTK depletion increased the expression of leukocyte-endothelial cell adhesion molecules, ICAM-1 (intercellular Adhesion Molecule 1) and VCAM-1 27. Therefore, we mainly investigate the role of MerTK in regulating inflammatory factors and chemokines in proteomics. **Figure 6E** and **6F** are upstream pathways with top 50 upregulated and representative downregulated signaling, respectively. Consistently with the findings in **Figure 5**, we found that, compared with WT group, *MerTK^-/-^* activates inflammatory factor MAP4K4 (MAPK4 kinase 4) while inhibits anti-inflammatory factors (e.g., IL-4, TGF-β1 and IL-10RA) and anti-inflammatory chemokine CXCL12. **Figure 6G** is the canonical pathway that shows activation of mitochondrial dysfunction and inhibition of EIF2 signaling, Eif3 and P70s6K signaling and protein ubiquitination pathway in *MerTK^-/-^* LCA compared to WT LCA. Next, we further analyzed the networks of activated signaling (e.g., JUN-jun proto-oncogene, FOS-fos proto-oncogene, AP-1 family-activator protein-1, and HIF1α-hypoxia inducible factor 1 subunit alpha, **Figure 6H**) and inhibited signaling (e.g., IL-4, IL4 receptor-IL4R, and STAT3,4-signal transducer and activator of transcription3,4, **Figure 6I**). Finally, we summarized the associated disease pathways based on -log (p value), showing that *MerTK^-/-^* may contribute to infection, brain dysfunction, heart dysfunction, peripheral arterial disease, sclerotic lesion, and artery occlusion (**Figure 6J**).

## Discussion

Efferocytosis represents a pathway for the clearance of apoptotic cells that is mainly performed by macrophages and immature dendritic cells 28. MerTK is the critical receptor for efferocytosis and determines the capacity of efferocytosis 29. *In vitro*, our findings provide persuasive evidence for the high ability of aortic ECs via MerTK to accomplish efferocytosis, and they indicate that d-flow inhibits MerTK expression and subsequently impairs EC efferocytosis. *In vivo*, *MerTK^-/-^* aggravates abnormal endothelial thickening and endothelia dysfunction, impairing endothelial efferocytosis and promoting atherosclerosis. Our findings provide another persuasive evidence for the role of MerTK in regulation of endothelial dysfunction and atherosclerosis. Our findings also show the feasibility for the setup acute atherosclerosis model combining with PCL-induced d-flow, AAV8-PCSK9 and MerTK gene knockout. Our observations may be helpful to open new avenues for potential therapeutic interventions designed to modulate EC efferocytosis in endothelial dysfunction and its associated cardiovascular diseases, such as atherosclerosis and vascular aging.

We performed big data analytics (epigenetic maps and tissue GTEx), RNA-seq and proteomics to find clues to unique pathways of MerTK in d-flow-mediated endothelial dysfunction and atherosclerosis, gaining insight into mechanisms by which endothelial MerTK restoration confer protection from impaired efferocytosis and atherosclerosis. Most importantly, our findings in big data analytics, RNA-seq and proteomics are consistent, showing that MerTK is sensitive to the changes of blood flow patterns and d-flow inhibits MerTK expression, leading to impaired EC efferocytosis and vascular dysfunction. Previous studies showed that phagocytosis of apoptotic cells by macrophages is impaired in atherosclerosis and atherosclerotic lesions further impairs efferocytosis, resulting in accumulation of apoptotic cells, secondary necrosis, and pro-inflammatory response 30-32. However, very few studies focused on efferocytosis in ECs and whether EC efferocytosis plays a role in endothelial dysfunction and atherosclerosis. It is also unknown whether blood flow patterns play a role in EC efferocytosis and the role of MerTK in this process. To answer these questions, *in vitro*, a step-flow chamber designed by us and an ibidi pump system were used to cross-validate our findings, which shows that MerTK is sensitive to shear stress and is significantly inhibited by d-flow. *In vivo*, PCL surgery for the acute d-flow induction was performed in WT and *MerTK^-/-^* mice. Our proteomics and immunostaining show that d-flow impairs engulfment and phagocytosis of red blood cells, and MerTK gene deficiency aggravates endothelial dysfunction in atherosclerosis. Mechanisms study by canonical pathways analysis show that mitochondrial dysfunction, EIF2 signaling, and protein ubiquitination pathway may play important roles in MerTK-mediated vascular dysfunction subjected to d-flow. It is note that the results of EIF2 downregulation is acquired by IPA with canonical pathways. Studies have shown that downregulation of EIF2 signaling promotes progression of atherosclerosis 33 that is consistent with our findings. As expected, the machine learning disease pathways show that MerTK gene deficiency is associated with occlusion artery disease, heart dysfunction and brain disorders. Interestingly, our proteomics data not only are consistent with the previous findings but also provide several novel mechanisms related to d-flow and MerTK. For the consistency, we found that MerTK gene deficiency activates MAPKs (MAPK1,3 and JNK) and pro-inflammatory cytokines (e.g., IL-1 and IGF1), while inhibits anti-inflammatory cytokines (e.g., IL-4 and TGF-β). For the novel pathways, several collagen-related signaling (e.g., Col14a and Col1a2), CD34, ASXL1, and other novel signaling mentioned in the section of results may be involved in MerTK-mediated endothelial function.

Two flow systems, a step-flow chamber and an ibidi pump System, were used to cross-validate the role of d-flow in regulating MerTK expression in HAECs. Our data shows that d-flow generated in a step-flow chamber has a similar effect on HAECs as OSS generated in an ibidi pump system. Consistent with the expression of endothelial dysfunction markers, increased pro-inflammation markers (NLRP3 inflammasome, TNF-α, and NF-κB) induced by d-flow accompanied with decreased expression of eNOS and anti-inflammatory marker TGF-β induced by OSS. For the possible mechanisms in d-flow mediated MerTK inhibition, it is known that d-flow induces inflammation response in endothelial cells 34. A large body of evidence shows that inflammatory factors (e.g. IL-1β, TNF-α, ox-LDL, and LPS) inhibit MerTK expression 35-38. Consistently, our data of Western blotting, immunostaining, RNA-seq and proteomics showed that d-flow induces pro-inflammatory response (e.g. increased expression of NF-κB, TNF-α, IL-1β and TLR4) and *MerTK^-/-^* aggravates d-flow-mediates pro-inflammatory response (e.g. activated MAPK signaling and IL-1R as well as inhibited IL-4 and TGF-β1). This indicates that inflammatory response plays an important role in MerTK inhibition and subsequent impaired efferocytosis in atherosclerosis. However, there are some limitations in our current study. First, it has been shown that MerTK is expressed in macrophages 39, vascular ECs 8,27, and vascular smooth muscle cells (SMCs) 40.

Although only aortic ECs are directly exposed to wall shear stress in the physiological conditions compared with macrophages and SMCs, it cannot differentiate which cell-expressed MerTK plays the most important role in d-flow-mediated atherosclerosis with *MerTK^-/-^* mice. Future study needs to be performed with MerTK specifically knockout in ECs to clarify the role of endothelial MerTK in atherosclerosis. Second, besides efferocytosis, MerTK also has many other functions in cardiovascular diseases, brain disorders and cancer, such as maintains the integrity of the endothelial barrier 41,42, acts as a transcription factor regulating dendritic cell differentiation 43,44, and regulates immune suppression in the tumor microenvironment 45. This indicates that MerTK may have double-edged sword functions in these human diseases 46.

In conclusion, we provide evidence for a relationship between d-flow, MerTK and endothelial efferocytosis and its implications in atherosclerosis. It is of note that aortic ECs have strong ability to perform efferocytosis, showing for the high engulfment of apoptotic Jurkat cells within 1 h in HAECs. In addition, aortic ECs may have higher efferocytosis ability than macrophages due to their bigger cell size, providing important translational clues that endothelial efferocytosis may be a promising therapeutic target for the treatment of atherosclerosis.
